# User-Independent Motion State Recognition Using Smartphone Sensors

**DOI:** 10.3390/s151229821

**Published:** 2015-12-04

**Authors:** Fuqiang Gu, Allison Kealy, Kourosh Khoshelham, Jianga Shang

**Affiliations:** 1 Department of Infrastructure Engineering, University of Melbourne, Parkville, Victoria 3010, Australia; a.kealy@unimelb.edu.au (A.K.); k.khoshelham@unimelb.edu.au (K.K.); 2 Faculty of Information Engineering, China University of Geosciences, Wuhan 430074, China; jgshang@cug.edu.cn

**Keywords:** indoor positioning, indoor location-based services, activity recognition, motion state, pressure derivative, feature selection, smartphones

## Abstract

The recognition of locomotion activities (e.g., walking, running, still) is important for a wide range of applications like indoor positioning, navigation, location-based services, and health monitoring. Recently, there has been a growing interest in activity recognition using accelerometer data. However, when utilizing only acceleration-based features, it is difficult to differentiate varying vertical motion states from horizontal motion states especially when conducting user-independent classification. In this paper, we also make use of the newly emerging barometer built in modern smartphones, and propose a novel feature called pressure derivative from the barometer readings for user motion state recognition, which is proven to be effective for distinguishing vertical motion states and does not depend on specific users’ data. Seven types of motion states are defined and six commonly-used classifiers are compared. In addition, we utilize the motion state history and the characteristics of people’s motion to improve the classification accuracies of those classifiers. Experimental results show that by using the historical information and human’s motion characteristics, we can achieve user-independent motion state classification with an accuracy of up to 90.7%. In addition, we analyze the influence of the window size and smartphone pose on the accuracy.

## 1. Introduction

Activity recognition is a vital technology that has been used or has the potential to be utilized for a wide range of applications, including transportation mode recognition [1], indoor positioning, navigation, location-based services, health monitoring, context-aware behaviors, targeted advertising, and mobile social networks [2]. Recent years have witnessed a significant increase in the variety of consumer devices, which are not only equipped with traditional sensors like GPS, camera, Wi-Fi and Bluetooth but also newly-developed sensors like accelerometer, gyroscope, and barometer. These sensors can capture the intensity and duration of the activity, and are even able to sense the activity context. This can help consumers assess their activity levels and change their activity behaviors to keep fit and healthy.

Equipped with a variety of sensors, smartphones are more attractive for activity recognition compared to on-body devices since they are ubiquitous, easy to use, and they do not disturb users’ normal activities [1]. Using these built-in smartphone sensors to sense user activity has been explored in the literature. GPS has been widely used for transportation mode classification and daily movement inference [3,4]. It can provide relatively fine-grained location information and the speed of movement, enabling the inference of if a user is driving or walking. Combined with a street graph or map, more high-level activities can be inferred. Sohn *et al.* [5] demonstrated the feasibility of using coarse-grained GSM data to recognize high-level user activities. By utilizing the fluctuation information between the radio and the cell tower, it is possible to distinguish whether a person is driving, walking or remaining at one place. Similar to GSM radio, Wi-Fi and Bluetooth radio can also be applied for activity recognition [6,7]. Microphone and camera are useful for activity recognition by offering acoustic and visual information, respectively [8]. Recently, there has been a significant interest in using accelerometer data for activity recognition [9,10,11], mainly because it can work both indoors and outdoors and has no need for extra infrastructure. Similarly, the gyroscope and magnetometer can enhance accelerometer-based activity recognition by detecting the change of smartphone’s orientation [12,13]. It is expected that in the near future, more types of sensors (e.g., air quality sensor) will be integrated into smartphones, which would provide richer context information.

A key component for activity recognition is classification algorithm. Lee *et al.* [11] used hierarchical artificial neural networks (ANNs) to recognize six daily activities (lying, standing, walking, going-upstairs, going-downstairs, and driving) according to the accelerometer signals. Lara *et al.* [14] developed a system, called Centinela, that can recognize five activities (walking, running, sitting, ascending, and descending) by combining acceleration data with vital signs. They showed that an accuracy up to 95.7% can be reached by using the additive logistic regression algorithm. Zhu and Sheng [15] proposed a two-step approach that fuses motion data and location information. In the first step, two neural networks were utilized to classify basic activities, followed by a hidden Markov model to consider sequential constraints. Then, the Bayes theorem was used to fuse location information and update the classified activities from the motion data. Kouris & Koutsouris [16] compared functional trees, naive Bayes, support vector machine, and C4.5 for physical activity recognition (standing, walking, jogging, running, cycling, and stairs), and concluded that the functional trees outperformed other classifiers. Pei *et al.* [12] used the least-squares support vector machine to recognize eight common motion states (static, standing with hand swinging, fast walking, U-turning, going upstairs, going downstairs, normal walking while holding the phone in hand, normal walking with hand swinging, and fast walking). Lester *et al.* [17] presented a hybrid approach combining the generative techniques with discriminative techniques to recognize different activities (e.g., sitting, standing, walking, jogging, riding a bicycle, driving car) by using wearable multi-sensor boards. The hidden Markov models were used to capture the temporal regularities and smoothness of activities. Hu *et al.* [18] presented a framework that uses skip-chain conditional random fields (CRFs) to recognize concurrent and interleaving activities. Liao *et al.* [19] applied hierarchical CRFs to extract and label a person’s activities and significant places based on GPS data and high-level context information.

This study focuses on locomotion activity (also known as motion state) recognition. Although a lot of research has been done in this field, some critical issues still need to be explored. Most existing motion-based recognition methods use only accelerometers and extract features from accelerometer readings. However, when using only acceleration-based features, it is difficult to differentiate varying vertical motion states from horizontal motion states. This is especially true when conducting user-independent classification. Generally, the acceleration characteristics of Walking and Upstairs/Downstairs for the same person are different but may be very similar between different users. The advent of the barometer sensor built in smartphones promises an effective solution to this problem. Barometer data have been used for floor-level indoor positioning in [20,21]. In this paper, we investigate the use of barometer data for motion state recognition. This paper has two main contributions: first, we propose a novel feature, called pressure derivative, obtained from barometer readings to distinguish vertical motion states in a user-independent manner; second, we develop a method to incorporate the motion state history in the classification of motion states and improve the classification performance.

The remainder of this paper is organized as follows. Section 2 introduces the key steps of our motion state recognition algorithm and defines the motion states of interest. Section 3 presents the partitioning method, definition of the proposed novel feature and the sequential forward feature selection method based on the least-squares support vector machine (LS-SVM). In Section 4, six commonly-used classifiers are briefly introduced, followed by the description of how motion state history and people’s motion characteristics can be used to improve classification accuracy. In Section 5, we evaluate the usefulness of the proposed feature, motion state recognition accuracy with and without historical information, and then analyze the influence of window size and smartphone pose on the classification accuracy. Section 6 concludes this paper and discusses the limitations of the study.

## 2. Overview of Motion State Recognition

**Figure 1:**
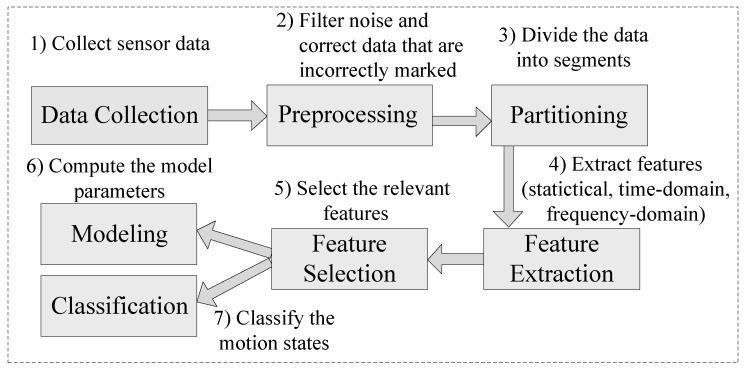
Flowchart of motion state recognition.

The main steps of motion state recognition are shown in Figure 1. At first, a variety of sensor data need to be collected, which can be done by developing a device-specific program or using some commercial applications. Then, these raw data are filtered to remove random noise during the preprocessing phase. After that, the partitioning is conducted to divide the continuous stream of sensor data into smaller time segments so that different features can be extracted, including statistical, time-domain and frequency-domain features. However, more features do not necessarily mean higher classification accuracy, hence it is important to use effective methods (e.g., filters and wrappers) to select the most appropriate features. Once relevant features are retrieved, a classifier can be trained and then used to classify new unlabeled data.

In this paper, we define seven types of motion states, namely Still, Walking, Running, Downstairs, Upstairs, DownElevator, and UpElevator, as shown in Table 1.

**Table 1:** Types of motion states.

No.	Motion States	Definition
M1	Still	The user carries a phone without any movement.
M2	Walking	The user is walking with a phone.
M3	Running	Horizontal running.
M4	Downstairs	Going down stairs.
M5	Upstairs	Going up stairs.
M6	DownElevator	Taking an elevator downward.
M7	UpElevator	Taking an elevator upward.

Generally, the outputs from the accelerometer vary at different poses, leading to a large variance in the features even if the user is staying in the same motion state. Therefore, we consider three common poses as shown in Table 2.

**Table 2:** Pose types.

No.	Poses	Definition
P1	Pocket	The phone is put in the trouser pocket.
P2	Holding	The user keeps a phone in his or her hand without swinging.
P3	Swinging	The user moves with a phone swinging in his or her hand.

## 3. Feature Extraction and Selection

The discriminative power of features selected has an important influence on the classification accuracy. More features do not necessarily increase the accuracy but raise the computational cost. Therefore, we use the LS-SVM-based sequential forward feature selection method to pick the relevant features. In this section, we start with the introduction of the partitioning method, then describe the types of features including the proposed feature, and finally present the feature selection method.

### 3.1. Partitioning

Typically, features are extracted from sensor data within a short time frame called a window. The window size has an influence on the classification results, and an appropriate window size is crucial for accurately recognizing user motion states in real time. A smaller window size may not precisely capture the full characteristics of motion states, while a larger one can introduce noise as it may contain multiple motion states [22]. Typically, a window of one second is often utilized for activity classification, which has been validated by prior work [23]. To better address the challenge, varying window sizes can be used simultaneously [24]. By computing multiple feature values within these windows, the optimal window size for each type of motion can be determined according to the classification performance using each feature value. However, maintaining multiple window sizes for each type of sensor data imposes a heavy burden on the devices, which may be impractical for applications that run on the smartphone.

**Figure 2:**
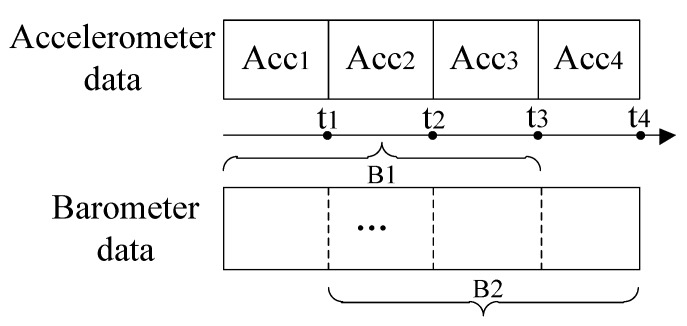
Partitioning and alignment of different types of sensor data.

Generally, different types of sensor data need varying segment sizes. For the accelerometer data, one or two seconds of segment size is appropriate to detect the change of acceleration. While for the barometer data, two seconds is too short to provide meaningful information. Therefore, we apply different segment sizes to varying types of sensor data. In order to combine the accelerometer data and barometer data for recognizing motion states defined, these two kinds of data are aligned according to their timestamps, as shown in Figure 2. For the accelerometer data, each segment represents a window of accelerometer readings. For the barometer data, each segment includes *k* windows of barometer readings where the value of *k* can be empirically determined. The window sizes for both accelerometer data and barometer data are the same, but the segment size of barometer data is k times than that of accelerometer data.

### 3.2. Feature Extraction

**Figure 3:**
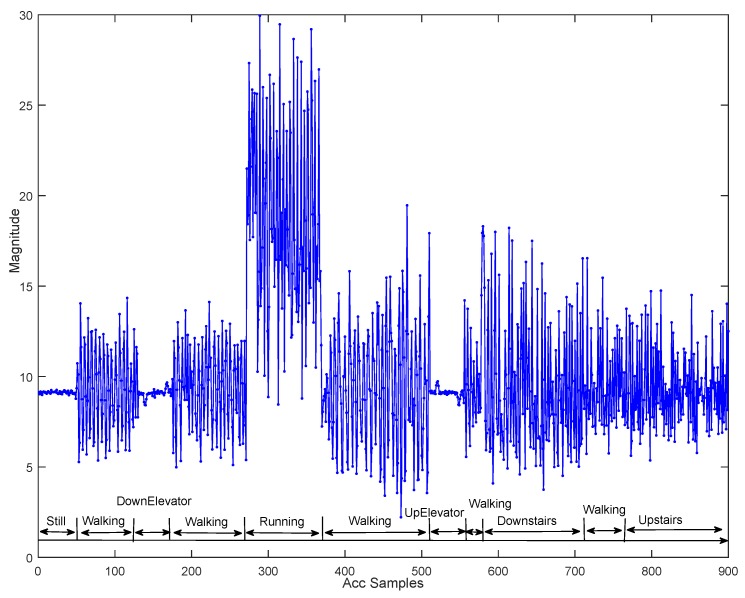
Acceleration magnitude of different motion states (holding pose).

There are lots of features that can be applied to activity recognition [25], including statistical features (e.g., Mean of acceleration), time-domain features (e.g., Zero-crossings of acceleration), frequency-domain features (e.g., FFT DC component of acceleration) and discrete-domain features (e.g., Dynamic Time Warping of acceleration). Most existing research projects only rely on the accelerometer to distinguish different activities. It is true that activities like Walking, Running and Still can be easily classified by using the accelerometer data. However, it is difficult to recognize in-elevator motion states with Still, and Stairs with Walking, especially when considering different users’ data. This is illustrated by Figure 3 giving the total acceleration magnitude of varying motion states, from which we can see that the characteristics for Walking and Stairs or for Elevators and Still are very similar.

**Figure 4:**
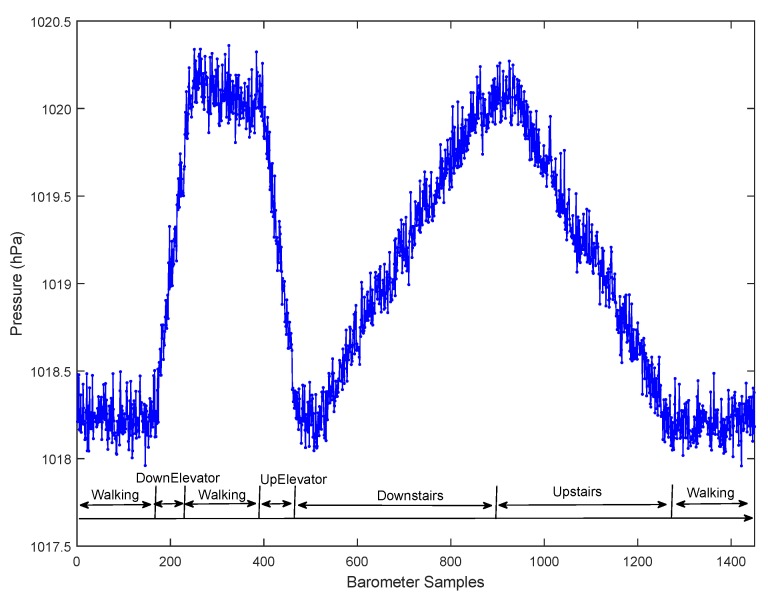
Comparison of pressure change between vertical and horizontal motion states.

On the other hand, pressure measurements captured by the barometer available on most smartphones today can help effectively distinguish those similar motion states. As shown in Figure 4, there is an obvious difference in the barometer readings between vertical and horizontal motion states. In order to precisely distinguish UpElevator/DownElevator, Downstairs, Upstairs from horizontal motion states, we define a novel feature called pressure derivative. In the following, we will give details about this feature.

Let $p_{k}$ indicate the $k_{th}$ segment of barometer readings, which is a vector including *n* barometer data points. Since it is observed that the readings in the barometer present a linear increase with slight random fluctuations when the user moves vertically, we can utilize the linear equation to fit these readings. Assume that the linear equation is $p^{\prime }=\dot{p}t+c$, where $\dot{p}$ is the pressure derivative to be solved for each segment of data and *c* is a constant. The objective is to find the best pressure derivative value for the equation which fits the data, namely

$${\hat{p}}_{k}=\underset{\dot{p}}{argmin}\sum_{j=1}^{n}{\left(p_{j}^{\prime }-p_{j}\right)}^{2}=\underset{\dot{p}}{argmin}\sum_{j=1}^{n}{\left(\dot{p}t_{j}+c-p_{j}\right)}^{2}\;\left(1\right)$$

**Figure 5:**
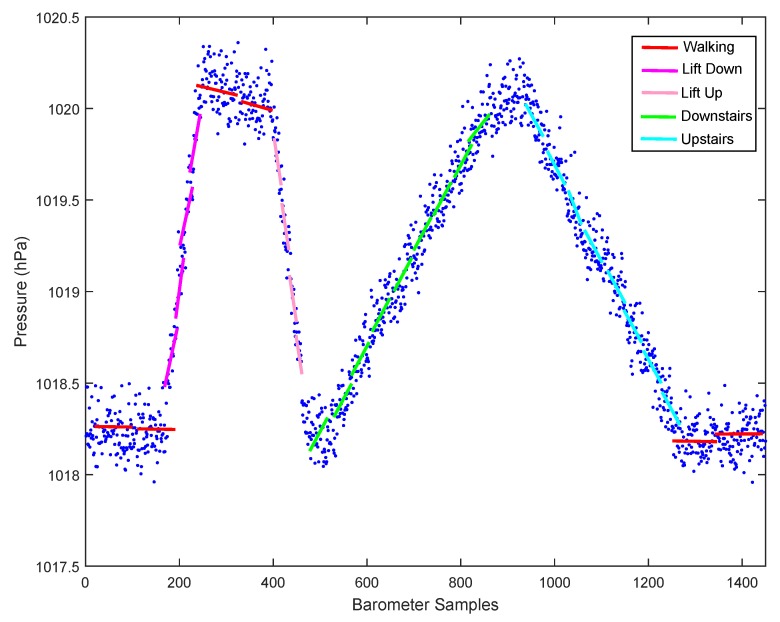
The pressure derivatives of different motion states.

Figure 5 shows that the pressure derivatives for the same motion state are similar while those for different states are significantly distinctive. The biggest advantage of this novel feature is its independence of users and poses, which means that it has good generalization ability and does not change with the user or smartphones’ poses.

### 3.3. Feature Selection Method

**Figure 6:**
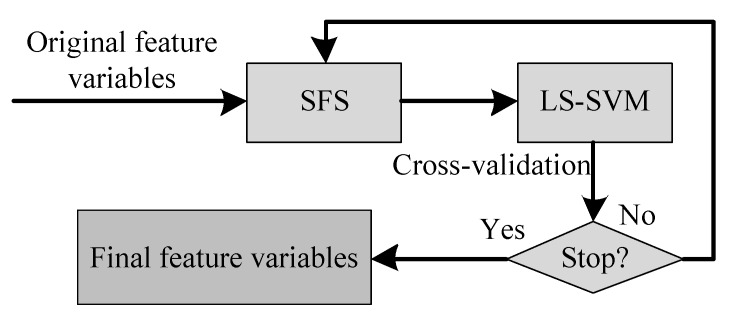
The LS-SVM-based feature selection method.

Feature selection is a vital process to select relevant features, which not only improves the recognition rate but also reduces the computational cost. In this study, we adopt the LS-SVM-based SFS method to achieve high classification accuracy, as shown in Figure 6. This algorithm starts from an empty feature set and iteratively adds one selected feature with maximum accuracy to the current feature set. The final feature variables are selected by evaluating the LS-SVM performance using cross validation.

## 4. Motion State Classification

### 4.1. Classifiers

In this study, we compare the performances of six classifiers, including decision trees (DT), linear discriminant analysis (LDA), K-nearest neighbors (KNN), Naive Bayes (NB), support vector machine (SVM), and least-squares support vector machine (LS-SVM). Here, we provide a brief introduction for these classifiers.

DT [26] classifies data by constructing a tree model. Each tree consists of nodes, branches, and leaves (corresponding to the target classes). The key to building a tree is selecting the best attribute used to split the considered nodes, which is mainly done by measuring the impurity of the data set, like information entropy and Gini index. LDA is a generalization of Fisher’s linear discriminant [27], which uses the class specific information to maximize the ratio of between-class scatter to that of within-class scatter. KNN is a non-parametric method for classification and regression, which classifies the testing data by finding the samples in the training set with a minimal Euclidean distance to the testing data. NB is regarded as a baseline in text classification [28] since it is easy to implement and is relatively effective. It simplifies learning by assuming the independence between features given class, which can work well when the features are independent [29]. SVM [30] was originally proposed for binary classification problems. The basic idea is to find hyperplanes to separate data points of different classes. By mapping the raw data points from the input space into a high dimensional feature space, the separability of the classes can be increased, thereby improving the classification performance. The LS-SVM is an improved version of SVM [31]. Instead of solving convex quadratic programming problems for the classical SVM, LS-SVM solves a set of linear equations by introducing a least-squares loss function.

### 4.2. The Method for Incorporating Motion State History in Classification

**Figure 7:**
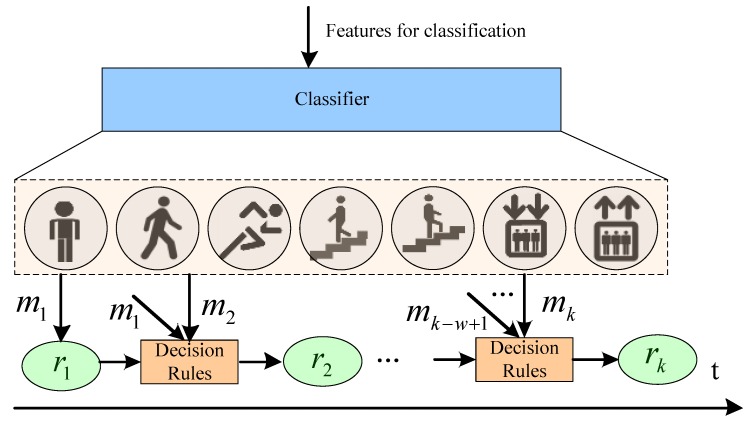
The classification method incorporating motion state history.

It is observed that user motion states are generally smooth and continuous, which means that users do not intermittently change their motion states and each motion state lasts for a certain period of time. This research makes use of the state history information and these characteristics of people’s motion to improve the classification accuracy of motion state recognition. The basic idea is shown in Figure 7, where $m_{k}$ represents the motion state recognized by the classifier at the step *k*, while $r_{k}$ is the final motion state. The intuition of this algorithm is to eliminate the jump phenomenon that the classifier reports varying motion states when the user stays in the same state.

The steps of the classification method incorporating motion state history are as follows. At the beginning, the initial motion state is recognized by the classifier, namely $r_{1}=m_{1}$, which is then fed to the Decision Rules module. According to the last final state $r_{k-1}$ and results from the classifier $\left\{m_{k-w},\cdots ,m_{k}\right\}$, the Decision Rules module determines whether to keep the last motion state ($r_{k}=r_{k-1}$) or transfer to a new motion state reported by the classifier ($r_{k}=m_{k}$). Specifically, the Decision Rules module outputs $r_{k}=m_{k}$ when the classifier reports consecutively *w* consistent results that are different from the last state $r_{k-1}$, otherwise it outputs $r_{k}=r_{k-1}$.

## 5. Evaluation

### 5.1. Data Collection

**Figure 8:**
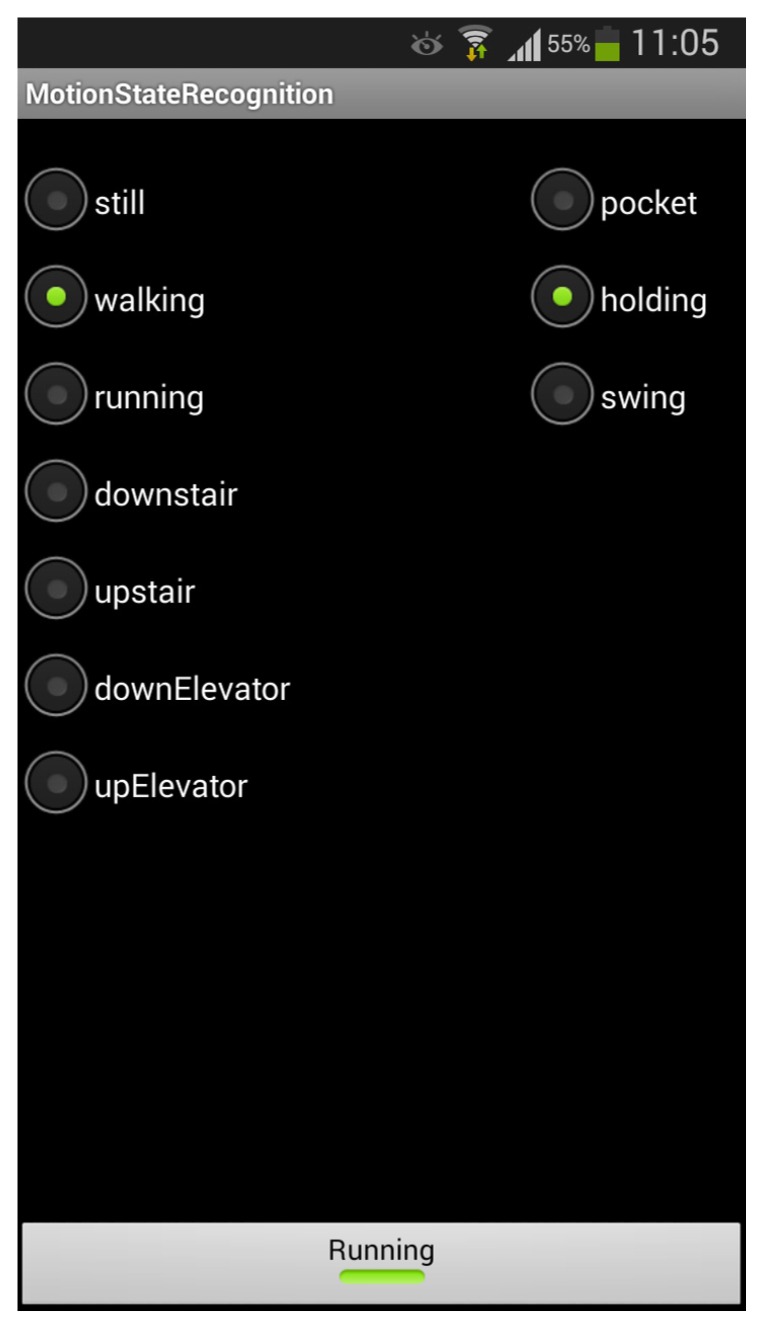
The app for recording sensor data, actual states and poses.

To collect the required data, we have developed an Android app that can record a variety of sensor data as well as actual motion states and poses, as shown in Figure 8. Both the accelerometer readings and barometer readings are recorded with their corresponding timestamps so that we can align them to jointly infer user motion states. The devices used are Samsung Galaxy SIII smartphones, which are equipped with the accelerometer, magnetometer, gyroscope as well as the barometer.

The experiments were conducted within the Infrastructure Engineering building located at the Parkville campus of the University of Melbourne and the surroundings of this building. It is a five-floor office building that includes typical structures like stairs and elevators. Eight participants were recruited to conduct the experiments. Each participant was asked to perform the defined seven motion states in all the three poses. Before the experiments, the participant needs to click the bottom button on the app to start logging data. When the smartphone is held in the hand, actual motion states and poses can be recorded by clicking the corresponding button. Errors in the actual states are allowed when recording the sensor data, which will be corrected during the preprocessing step. When the phone is put in the pocket, the app records only the sensor readings and the actual poses, while the corresponding actual motion states are manually labeled after the data acquisition.

### 5.2. Performance Evaluation

The evaluation metric we used is accuracy [32]. Let $N_{s_{i}}$ indicate the total number of sensor segments associated with a predefined motion state $s_{i}$, and $TP_{s_{i}}$ represent the number of correctly classified segments for the corresponding state. Then, the accuracy can be expressed as,

$$accuracy=\sum_{i=1}^{\left|s\right|}\frac{TP_{s_{i}}}{N_{s_{i}}}\;\left(2\right)$$

The toolboxes we used include LS-SVMlab [33], Weka [34], Feature Selection [35] and Matlab Statistics and Machine Learning toolbox.

To generalize the classification, the data collected by these eight participants are separated into two groups with each including four persons’ data. The data from the first group are used for training, while data from the second group are used for testing. This guarantees that the classification is independent of the users. Initially, 66 features were extracted, including 57 from the accelerometer readings and 9 from the barometer readings, but only 7 were used at the end after conducting the LS-SVM-based feature selection. The segment size for the accelerometer was 64 (corresponding to 2 s) without overlap while for the barometer it was 64 × 5 with 80% overlap. The overall number of data segments was 10,239, in which 5140 data segments were used for training (first group’s data), and the remaining 5099 were used for testing (second group’s data). The number of segments for each motion state is shown in Table 3.

Next, we begin with analysis of the effectiveness of the proposed feature and accuracy of user-independent classification, and then present the influence of the window size and smartphone pose.

**Table 3:** Number of segments for each motion state.

Motion States	Number of Segments
Still	1471
Walking	5193
Running	801
Downstairs	1136
Upstairs	1079
DownElevator	275
UpElevator	284

#### 5.2.1. Effectiveness of Pressure Derivative Feature

**Figure 9:**
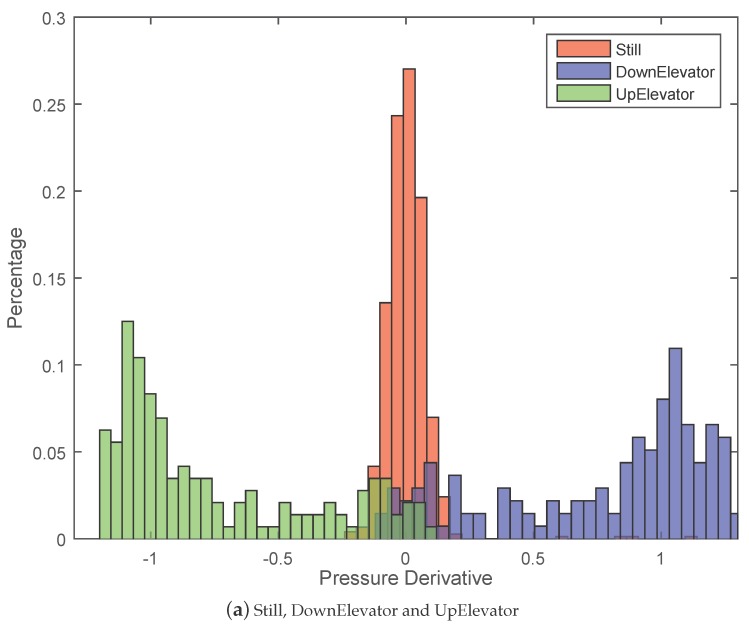

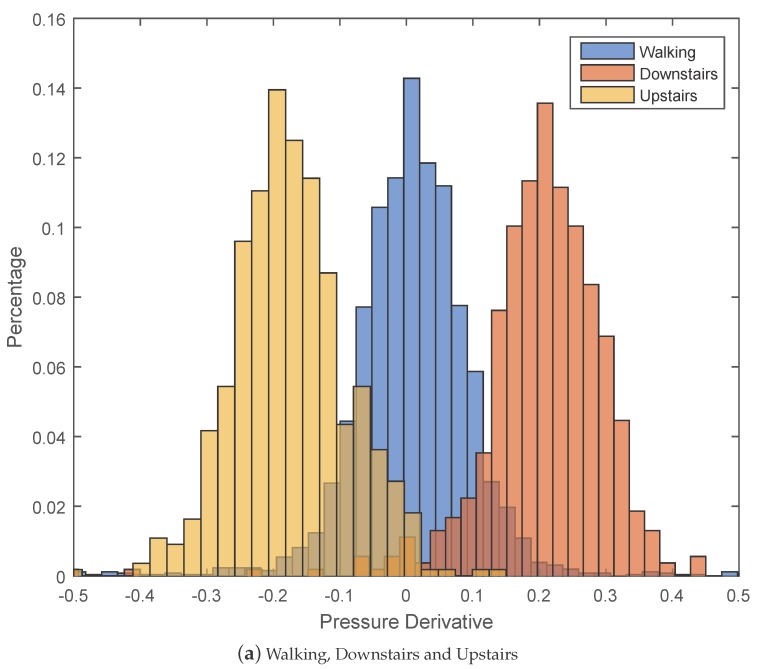
The distinguishability of the pressure derivative feature.

Figure 9a,b show the effectiveness of the pressure derivative for distinguishing varying vertical motion states from horizontal motion states. We can see that the values of pressure derivative for different motion states fall into varying intervals. Specifically, the value of pressure derivative for Still mainly falls into the interval [−0.15, 0.15], while the values for DownElevator and UpElevator fall into the intervals [0.2, 1.2] and [−1.2, −0.2], respectively. The pressure derivative value for Walking ranges within [−0.1, 0.1] while the values for Downstairs and Upstairs mainly fall into [0.1, 0.3] and [−0.3, −0.1], respectively. Although there is a little overlap area between In-elevator and Still, and between Downstairs, Upstairs and Walking, Figure 9a,b still demonstrate that using the pressure derivative feature can effectively separate In-elevator state from Still, Walking from Downstairs and Upstairs.

In addition, the effectiveness of the pressure derivative can be justified by the result of the LS-SVM-based SFS method. The final 7 types of features selected are shown in Table 4, which also demonstrates the order of each feature being selected. These features selected include variance of the total acceleration, pressure derivative of barometer data, root mean square of the horizontal acceleration, skewness and range of the vertical acceleration, pressure difference between two windows of barometer readings, and maximum value of the vertical acceleration. The proposed novel feature (pressure derivative) was chosen in the second place, which shows its effectiveness in motion state recognition. In addition, we compared the classification accuracy of LS-SVM with and without the pressure derivative feature. It shows that removing the pressure derivative from the set of features results in a reduction of classification accuracy ranging from 3.0% to 7.6% for different (vertical) motion states.

**Table 4:** Final features selected by the LS-SVM-based SFS.

Step No.	Feature Name
1	variance of the total acceleration
2	pressure derivative of barometer data
3	root mean square of the horizontal acceleration
4	skewness of the vertical acceleration
5	range of the vertical acceleration
6	pressure difference between two windows of barometer readings
7	maximum value of the vertical acceleration

#### 5.2.2. Motion State Recognition Accuracy

**Figure 10:**
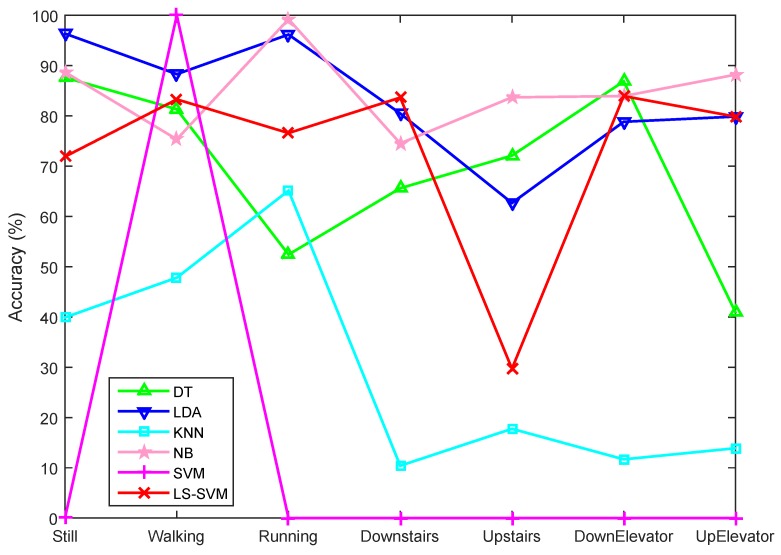
Classification accuracies of different classifiers with all features.

**Figure 11:**
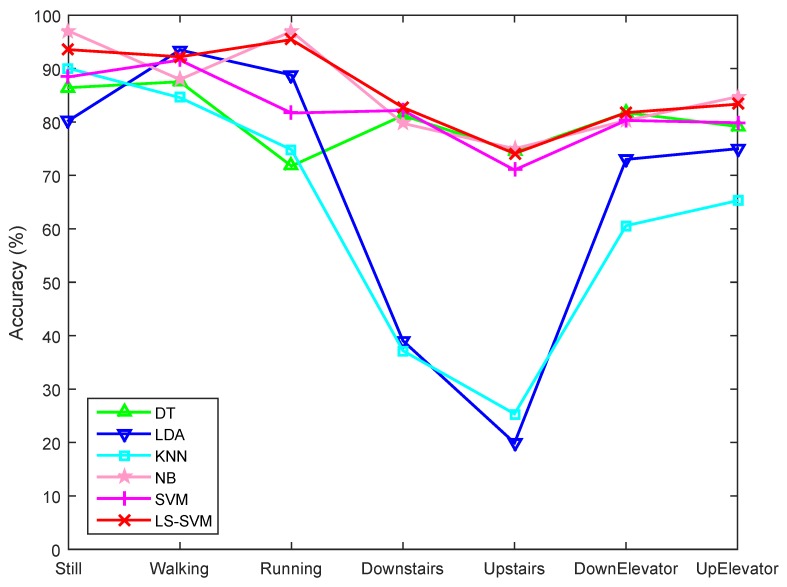
Classification accuracies of different classifiers with features selected by the LS-SVM-based feature selection method.

The recognition results of the four volunteers in the second group are demonstrated in Figure 10 and Figure 11, from which we can see that including more features does not mean higher classification accuracy. In Figure 10, it is interesting to note that when using all features the SVM classifier classifies all data segments as belonging to the Walking motion state. This is partly due to the presence of weak features, which are not sufficiently distinctive for other motion states, and partly due to the uneven distribution of the data segments, where Walking has the largest number of data segments (see Table 3). Figure 11 shows that feature selection can overcome this problem. By selecting a subset of features all classifiers, except for LDA, perform better, while LS-SVM shows the best performance.

**Figure 12:**
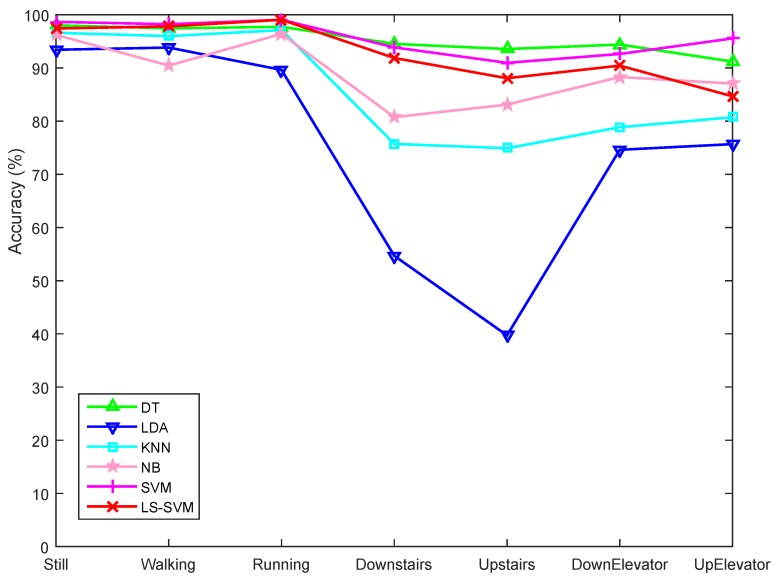
User-dependent mean training accuracies for the first group. The features used were selected by the LS-SVM-based feature selection method. The data of each person in the first group were used for both training and testing.

Figure 10 and Figure 11 also reveal that horizontal motion states of the subjects have higher classification accuracies than those involving vertical motion. Still, Walking, and Running were generally better recognized than other states. Downstairs and Upstairs have the lowest recognition rates since they share some common characteristics with the Walking state. One reason for this is that we conducted user-independent classification, which means that the training data and testing data were from different data groups. This can be explained by comparing the training accuracies (Figure 12) with user-independent testing accuracies (Figure 11), where we can see that the accuracies in Figure 12 are much higher than those in Figure 11. Generally, the acceleration characteristics of Walking and Upstairs/Downstairs for the same person are different, but may be very similar between different users, which may further result in an increased recognition error. Another reason is that, although we used the barometer readings to help recognize vertical motion states, which are effective when users move a certain distance in vertical direction, it is still difficult to accurately recognize vertical motion states during a certain time period after a transition. For example, when users transfer from Walking to Upstairs/Downstairs, the subsequent several states after the transition are more likely to be recognized as Walking since the pressure feature is useful only when a sufficiently long vertical distance is traveled.

**Figure 13:**
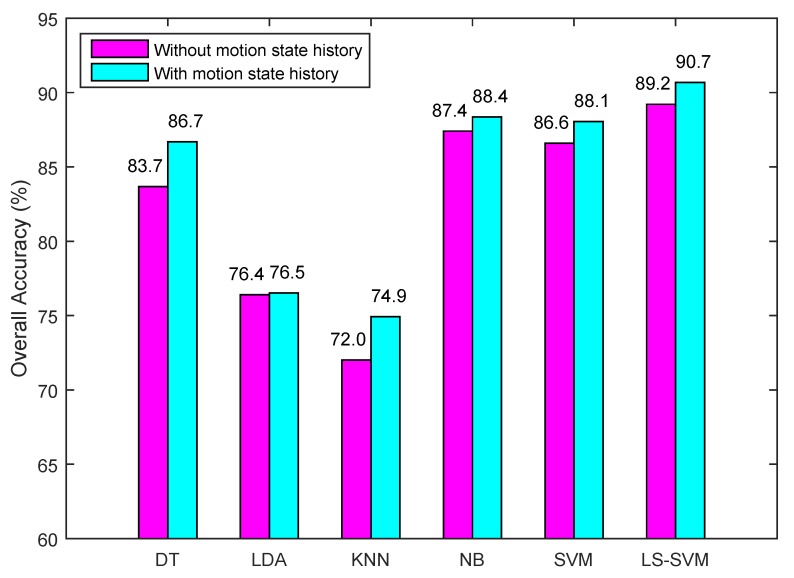
Overall accuracy comparison between with and without motion state history.

The effect of using the motion state history was evaluated by comparing with and without incorporating state history. From Figure 13, we can see that incorporating state history increases the recognition rate by 1.6% on average. Specifically, the biggest accuracy improvement is in the DT classifier where it was improved by 3%. The improvements for SVM, LS-SVM, and KNN stay in the second place, followed by NB, while LDA has the least improvement in the classification accuracy. Overall, the highest classification accuracy was achieved by the LS-SVM. NB stayed in the second place, which was followed by SVM and DT. The other two classifiers (KNN, LDA) had lower classification accuracies because LDA usually needs to take more features as input for achieving a relatively high accuracy while KNN is not suitable to deal with complex classification problem.

#### 5.2.3. Influence of Window Size

The influence of different window sizes on the classification performance was analyzed, as depicted in Table 5. The sampling rate of the accelerometer used is about 32 Hz, which means that it reports 32 samples every second. We compared the performance of four different window sizes with 50% overlap and with no overlap, respectively.

**Table 5:** Accuracies of varying window size and sliding offset.

Window Size	Offset	Overall Accuracy					
		DT	LDA	KNN	NB	SVM	LS-SVM
	0	72.64%	75.18%	69.28%	77.28%	79.18%	79.66%
32	16	72.83%	75.36%	69.56%	77.44%	79.61%	79.81%
64	0	83.68%	76.41%	72.01%	87.41%	86.64%	88.63%
64	32	83.75%	76.60%	71.88%	87.10%	86.89%	87.21%
128	0	86.46%	74.80%	71.70%	87.44%	88.26%	88.54%
128	64	85.68%	76.47%	71.94%	88.72%	88.42%	86.43%
256	0	86.24 %	75.55%	70.68%	86.79%	87.66%	88.29%
256	128	83.89%	77.98%	70.83%	88.21%	87.69%	88.10%

Generally, the performances of all classifiers improve with increasing the window size until the window size reaches up to 64 where improvements are more significant. After this, increasing the window size does not necessarily enhance the accuracy but may cause some problems. For example, a too large window size would ignore states that only last for a few seconds or result in higher latency in motion state recognition. In addition, there is no significant difference between the partitioning method with and without overlap. Therefore, choosing a proper window size requires that one considers accuracy, real-time capability, and the ignorance for short states. In this paper, we adopted the window size of 64 as the segment size of acceleration without overlap and 64 × 5 with 80% overlap for the barometer data.

#### 5.2.4. Influence of Smartphone Poses

To evaluate the influence of smartphone poses on the classification accuracy, we conducted both pose-specific and overall experiments using the LS-SVM classifier, which usually gives the highest accuracy. For the pose-specific experiment, the training data and testing data were collected in the specific pose (Pocket, Holding or Swinging). Similarly, four person’s data acted as training data while the other four person’s data as testing data. For the overall experiment, data collected at all the three poses were considered. Table 6 shows the results, from which we can see that holding the phone in the hand had higher accuracy than putting it in the other two poses, which is almost equal to the overall case.

**Table 6:** Accuracy comparison of LS-SVM for varying phone poses.

Poses	Overall Accuracy of LS-SVM
Pocket	86.67%
Holding	89.09%
Swinging	87.33%
Overall	89.21%

## 6. Conclusions

We presented a method for user-independent motion state recognition using sensors built in modern smartphones. A feature called pressure derivative was proposed to distinguish vertical motion states from horizontal ones. We also described that the performance of commonly-used classifiers for motion state recognition can be further improved by considering state history and people’s moving characteristics. In addition, the influence of both the window size and smartphone pose on the classification accuracy was analyzed.

However, there are still several limitations. Although the proposed pressure derivative is useful for recognizing vertical motion states, there is a delay to accurately recognize Downstairs/Upstairs when users transfer from Walking state. It is the same case when users transfer from Still to DownElevator/UpElevator. This is because barometer readings can be useful for vertical motion state classification only when users travel a certain distance in vertical direction. This results in the relatively low classification accuracies of vertical motion states compared with horizontal motion states. This problem is expected to be solved by considering information from other sensors or environment. For instance, when a user enters an elevator, there is a significant decrease in the Wi-Fi signal strength, which can be combined to judge whether users are in the elevator. In addition, we do not consider less common poses. For example, a person in a Still state might swing the phone in his/her in hand as in a Walking state. This kind of uncommon pose may be addressed by considering the change of Wi-Fi fingerprints when users stay in Wi-Fi covered areas. In addition, the transition from state to state and that from pose to pose is currently not considered in this study.

In future work, we anticipate that much more complex activities like studying at home and shopping can be recognized so as to better understand people’s living behaviors and provide contextual services. We will also investigate the role of motion state recognition in improving localization accuracy in indoor environments.
